# Modelling product lines diffusion: a framework incorporating competitive brands for sustainable innovations

**DOI:** 10.1007/s12063-022-00260-0

**Published:** 2022-05-11

**Authors:** Deepti Aggrawal, Adarsh Anand, Gunjan Bansal, Gareth H. Davies, Parisa Maroufkhani, Yogesh K. Dwivedi

**Affiliations:** 1 University School of Management and Entrepreneurship, Delhi Technological University, Delhi, India; 2 Department of Operational Research, University of Delhi, Delhi, India; 3 School of Management, Swansea University, Swansea, UK; 4 The University of Waikato Joint Institute, Zhejiang University City College, Hangzhou, PR China; 5 Zhejiang University City College, Hangzhou, People’s Republic of China; 6 Emerging Markets Research Centre (EMaRC), School of Management, Room #323, Swansea University, Bay Campus, Fabian Bay, Swansea, SA1 8EN Wales UK; 7 Department of Management, Symbiosis Institute of Business Management, Pune & Symbiosis International (Deemed University), Pune, Maharashtra India

**Keywords:** Competitive brand, Customer behavior, Diffusion modelling, Process disruption, Product line, Social change

## Abstract

Understanding of consumer behavior, their changing demands due to increase in social interactions and communications, adoption of latest technologies over existing products have always been a set of fundamental activities for the firms. Keeping the objective of minimum process disruptions and discouraging product proliferation, firms always endeavor to match heterogeneous demands of consumers by emphasizing on the product line. Also, with globalization, rivalry amongst firms has reached a next level. Brands are trying to capture the market by coming up with various combinations of new product mix. Amongst various attributes of product mix, product line has helped firms to attract new potential buyers to a significantly good extent. Therefore, in today’s cutthroat competitive scenario, the concept of product line provides an opportunity for a firm to provide same kind of products with some variation at an altered pricing. The objective of this study is to understand how customers behave (with so many options) and deviate from one product to another product (within and outside the brand). All the possible customers’ shifting combinations that might impact the overall sales of product are captured through the proposed model. A mathematical innovation diffusion model is developed that is motivated by the concept of Bass model and multiple generational diffusion models. This modelling framework describes the scenario of competitive brands that offer multiple products in a marketplace and observing the shifting behavior of the customers and predict the sales when product lines are available. Validation of the model has been done on real-life sales data sets for automobiles industries of two different brands i.e., Hyundai and Maruti Suzuki. The importance of this study is to deliver a solution to the manufacturers that how consumer shifts from one brand to another brand. Therefore, it is imperative for the companies to develop such a product that would lead to customers’ loyalty towards the brand.

## Introduction

A market comprises a blend of different items which are being created by numerous manufacturers and utilized by different customers. An accessible item in the market assumes distinctive parts for both manufacturers and customers; where at one hand, producer’s sole responsibility is to generate revenue and build trust by satisfying customer’s needs, customers on the other hand use products to convert their needs into satisfaction (Crawford and Benedetto 2006; Hsiao et al. 2009). All manufacturers put their best available resources to attract customer for a longer period of time. In today’s competitive environment it becomes difficult for the manufacturer to keep the customer with them until and unless a company comes up with some new marketing strategies. Moreover, firms develop strategies that are equipped with less cost but embedded with a qualitative product (Gupta et al. 2020).

With the advent of globalization which has provided a lot of opportunities for both producers and customers now, it becomes more competitive for the producers. There are many companies available which are producing almost identical products with similar attributes at some different prices, for example, Maruti Suzuki and Hyundai are manufacturing Swift and i10 cars respectively, which have similar characteristics with some different prices. Competition depends upon various factors such as government, ease of doing business, type of market, etc. These days impure competition is more prevalent, where many manufacturers are producing similar products with different functions at different prices. Competition varies with product to product. Competitors can be classified as i) *direct competitors* are the manufacturers who are producing almost identical product in the same category. For example, Maruti and Hyundai are two well-known brands which can be called direct competitors in the field of manufacturing cars as a product and four-wheelers as a category; ii) *indirect competitors* are the manufacturers who produce a related product to some extent within the same category. For example, for Maruti manufacturer which produces cars as a product, an indirect competitor can be American automobiles brand that produces Jeep as a different product; iii) *replacement competitors* are the producers who deal in totally different products and different category. For example, any two-wheeler manufacturer which does not produce 4-wheeler cars can be considered as replacement competitor for the car producing company.

**Fig. 1:**
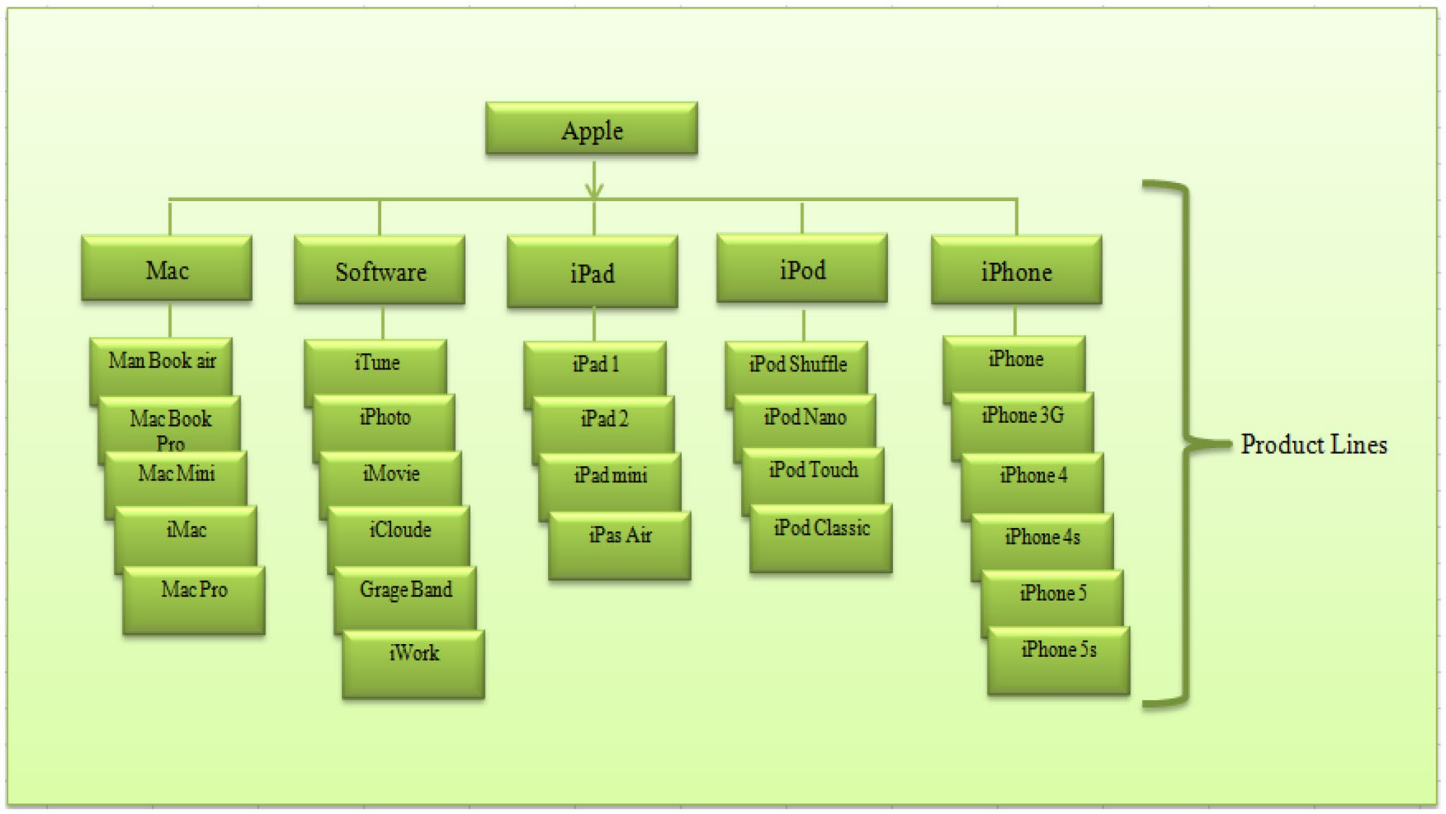
Apple and its Product Line (Bansal et al. 2016)

One of the major objectives of any company is to generate more revenue and have an upper edge in this competitive market. It is very difficult to survive in the market with just one product as demands are getting dynamic in nature and the technology is also increasing at a much faster rate. Hence, competition brings the necessity to come up with other updated products as well. Therefore, many brands produce either similar goods or goods which are altogether new to the world. These days’ companies spend a lot in research and development so that they can hit the market with the right product at the right time and meanwhile keep on updating their already existing products in the market, which helps them to target some new segment of the market. In the competitive environment, when a company adopts a strategy to come up with the various types of products, then it is said to be a product mix which refers to the total number of product lines that a company offers to its customers and splitting of products into groups is termed as a product line. A product line is a group of related products under a single brand sold by the same company. Companies sell multiple product lines under their various brands. Companies often expand their offerings by adding to existing product lines, because consumers are more likely to purchase products from brands with which they are already familiar. For example, using Fig. 1, a very well-known company Apple Inc. (2021) which attracts audience in all over the world by providing new and high technology not only in one type of product. But it is working on the strategy of product mix i.e., a mixture of products of Operating Systems, Software, Tablets, MP3players, and Mobile phones. And, they have defined them with a new name as Mac, Software, iPad, iPod and iPhone respectively. These all are 5 different product lines of Apple Inc. by which they are generating satisfaction among customers.

In a particular product line, there might be different products available and different versions of the same product which is termed as “Multi-generational” products. A multi-generational product is a product that comes periodically with some improvisation in the existing product in terms of advanced technologies be it software or hardware and new features such iPhone-13 is the multi-generational product for iPhone-12. Hence, multi-generational products are the company’s strategy to be in the competitive market (Agarwal et al. 2017; Gupta et al. 2019). Different types of product line can be understood by means of Fig. 1. Software product line consists of a set of different varieties of software which are based on different clusters i.e. for downloading audios, music, television stories, etc. iTune application has been developed, whereas for the purpose of manipulation of the digital photographs, iPhoto has been build up, similarly for different usage and work they have developed different applications and its related software. This can be comprehended as different "product types" in the single product line which is measured as the length of a product line i.e. software product line has 6 lengths of different product types. But in case of iPhone product line, "iPhone 4" and "iPhone 4 s" and "iPhone 5" and "iPhone 5 s" are the example of multi-generated products which increases the depth of a particular "product type" within a product line.

Rest of the paper has been structured as follows: - Sect. 2 provides an overview of the relevant literature. Modeling framework based on competitive factor when more than one brand along with their product line exists in the marketplace has been discussed in Sect. 3. In Sect. 4, a numerical illustration based on automobile industry dataset has been demonstrated. Results have been interpreted and discussed here along with the significance of this study for managers i.e. Managerial Implication. The future scope and limitations of the work have also been presented in the same section. The study has been concluded in Sect. 5.

## Literature review

This section discusses about a brief introduction of concept of Diffusion modeling, research motivation and contribution of this study.

A new product or an innovation is either accepted or rejected by the customers (Anand et al. 2018b, 2019; Aggrawal et al. 2021). Therefore, adopting behavior of the adopters depends not only upon the word of mouth but also on the various attributes such as quality, promotions and price. In the pioneering work by Bass (1969) and in many of its extension-based modeling, internal factors (i.e. coefficient of $p$) and external factors (i.e. coefficient of $q$) have always been focused in determining the growth of the sales but there are other factors also available such as competitive scenario that affect the sales growth. A set of propositions have been developed by (Robertson and Gatignon 1986) that competitive environment of supply side and the competitive environment of adopting side both affect diffusion of the new product (Zhang et al. 2020). Therefore, in this modeling framework we have not only incorporated the basic factors of external and internal aspects but also examined the sales pattern in the presence of the other products which are in the competition with a particular product.

This well-established bass model (Bass 1969) is based upon hazard function that states the probability of purchasing a new product by a new purchaser at time $t$ given that no purchase has been made yet. Bass model has been widely accepted and applied in many fields like *multi-generational of a new product*: To maintain the interest of the existing customers towards product and to capture the new market, companies always come up with its newer and updated versions that have launched on timely basis (Adamuthe and Thampi 2019). This concept is known as multi-generational of a product. Norton and Bass (1987) had introduced this concept of successive generations of a product and then further extensions have been made that has great impact in the field of marketing (Aggrawal et al. 2015; Lieberman 1987). Mangano et al. (2019) used Bass model as a basis to study the dynamics of diffusion of an innovative ICT product for City Logistics Management. *Product Services*: Under this field, instead of tangible product, intangible products (i.e. Services) have been taken into consideration and proposed various mathematical modeling (Libai et al. 2009). Here, we are focusing on the concept of the Product Line of a particular brand that has been explained in next section.

Many models (Bass 1969; Anand et al. 2014, 2018a; Singhal et al. 2019, 2020) have been proposed in the past but they are specifically based on single product present in the market and moreover they do not explicitly talk about the concept of product line (Walsh et al. 2020). Lately, mathematical formulations for finding the best marketing mix for each product in a product line that consist the factors like aggregate product group marketing mix, product interdependency, and competitive brand effects have been discussed (Xu et al. 2015; Malhotra et al. 2019). Rivalry in the multigenerational product using Product line has been presented by (Brander and Eaton 1984). Furthermore, the concept proposed by (Wilson and Norton 1989) discuss regarding optimal timing for entry for a product line extension.

The concept of Product lines becomes very interesting to study when there is extremely high competition in the market. In such a market, several brands are actively participating, and they are giving tough competition to each other (Sinha et al. 2020). This provides a variety of choices to the customers and this phenomenon helps customers to choose best product from a wide range of similar products but with some different characteristics (Brander and Eaton 1984; Wilson and Norton 1989; Bansal et al. 2016; Ismagilova et al. 2017, 2020). It is difficult for the firms to make decisions related to launching a new product by extending an existing product line or by adding a new product line. Decisions related to product lines are difficult because they are dependent on many other products and cannot be analyzed individually. Urban (1969) has discussed a mathematical model that is useful to analyze the implications of interdependency among the products while making marketing strategies. The key objective to sustain in the market for the firm is to satisfy diverse demands of heterogeneous sets of customers that lead to product proliferation. Though products are similar but provides different satisfaction levels to different customers because of price variations. Kekre and Srinivasan (1990) have discussed about when there is a need to broaden the product line and how it is imperative to add a new product in existing product line by comparing market share benefits and cost in developing new and differentiated products. Michalek et al. (2006) provided a solution in optimizing design of product line by combining existing models with addition of quantitative models related to investment in marketing and product allocation. Marketing and engineering are distinct but require a close coordination in the designing of product line. A method corresponding to product line optimization has been developed by Luo (2011) that addressed the interrelated issues between engineering and marketing by considering important factors from both domains simultaneously. On a similar aspect, Michalek et al. (2011) have presented a product line optimization methodology that considers consumers’ preferences, constraints based on engineering domain and determine optimal number of products require in a product line. Bertsimas and Mišić (2019) have also provided an integer programming based solution for a product line design. Products can also be differentiated in terms of green and non- green products, Shen et al. (2019) have developed a product line design that distinguish the quality among such two differentiated products. In literature the concept of the product line has been discussed majorly in terms of its design keeping the cost and the customer preference in the mind.

Higher competition brings efficiency in terms of providing better quality in products and services. Companies define new strategies regularly by keeping customers’ needs on top priority that helps them in maintaining a strong relationship with the customers (Saeed et al. 2020; Shareef et al. 2016). Therefore, it becomes necessary for a brand to have the idea about the diffusion behaviour of the product in the market because it is competing with the products of the same brand and other brands as well (Knauber et al. 2002; Kapoor and Dwivedi 2020; Thompson 2020; Singh et al. 2015; Cao et al. 2019; Kwarteng et al. 2020; Mirzaei et al. 2021). In other words, *competitive factor* can act as a perfect blend of economic psychology and marketing science (Qian and Soopramanien 2014; Talukder et al. 2020; Wang et al. 2021). Understanding consumer behavior also goes a long way in product acceptance and sustainability (Schallehn et al. 2019; Nilashi et al. 2020; Dong-Young et al. 2020). Firms are providing differentiated products by minimizing the process disruption keeping as a constraint and fulfilling their diversified demand. But the gap that has hardly been addressed is; understating the switching behavior or response of the customers when a product competes with its own and its competitive products. Therefore, it becomes difficult and interesting to predict the deviating nature of customers in the presence of many brands competing along with itself in the presence of product line.

The objective of this paper is to study the influence of competitive factor that directly affects consumer’s behavior such as purchasing, saving, brand choice, etc. and induce consumers to deviate from one brand to another brand in the environment of impure competition with direct competitors. Also after using the derived information from the analysis, sales have been forecasted for different products of different product lines. By incorporating the concept of the product line for any brand helps them to achieve their goals in terms of more revenue and customer satisfaction as multi-generation is an on-going process which keeps on attracting customers (Oliveira et al. 2019). Another major benefit of using product line is, when a product has reached the last stage of its life cycle that is in the declining stage, and then at the same time there are other products available in the market of that product line which helps in avoiding loss of customers (or business) from its competitors (Kamolsook et al. 2019). This section discusses the methodological approach of studying shifting behavior of the customers from one product to another product and predict the sales for all the products that are competing. The problem definition, assumptions, notations, and model development are specified below.

This study is discussing a scenario where many brands are competing with different brands and allow to launch a similar product. This product competes with all other products that already exist in the market. ***Multiple generation (Bansal et al.*** 2021***) is defined as a specific case of product line***. Overall, the main contributions of this paper have been described below:

- In this study emphasis has been given to the ***customers*** who are the main actors in the field of marketing. Success of any new offering, brand all depends upon the choice of the customers. Therefore, for the firms it becomes significant to know their customers and their responses towards new offering.
- Another factor that has been address in this study is ***competition***. Competition brings the thrust of being the best brand in the marketplace and that leads to new and innovative ideas that corresponds to social change by emerging new demands.
- The concept of the product line has hardly been integrated with the innovation diffusion modeling. Using the above two factors, this paper has developed a mathematical framework that observe the customers’ behavior and predict the sales of the products that are coexisting with their competitive products.
- This study would help firms to analyze which products are being preferred by their own existing customers and at what rate new customers are being influenced by their new offerings.

## Mathematical modeling

### Problem description

This paper describes about the market where different products are available for a customer. They are different in terms of price, features, appearance, and brand names. Here, a customer has many choices to choose the best product from them. Therefore, changing behavior of customer may impact the sales expectations of manufactures. Therefore, this present study aims to formulate a mathematical formulation that helps to identify which product has taken the market and which product has lost its customers due to high competition.

The modelling framework is based upon some assumptions which have been listed below:

### Assumptions

- The number of potential customers for each product line, *m* is a constant.
- There are two broad categories of potential buyers i.e. Purchasers and Deviators.
- Competition between the two brands along with their multi-products coexists has been considered.
- Only one unit is being purchased by any new buyer. No repeat purchase is possible as we have taken cars as our products which happen to be durable products.
- Product lines are comparable to each other of different brands.

### Notation

**Table:** 

${\lambda }_{j}$	Rate that classifies overall potential customers of one brand into number of distinct products available
${m}_{i}$	Potential adopters of the ${i}^{th}$ brand for single product line i.e. all combined possible customers of one brand
${F}_{ij}(t)$	Cumulative likelihood of purchasing product of ${j}^{th}$ product belonging to the ${i}^{th}$ brand
${p}_{ij}$	Coefficient of innovation of ${i}^{th}$ brand of ${j}^{th}$ product
${q}_{ij}$	Coefficient of imitation of ${i}^{th}$ brand of ${j}^{th}$ product
${\alpha }_{k}$	Proportion that deviates from H_P1 product to other products
${\delta }_{k}$	Proportion that deviates from H_P2 product to other products
${\rho }_{k}$	Proportion that deviates from M_P1 product to other products
${\gamma }_{k}$	Proportion that deviates from M_P2 product to other products
$i$	Represents number of brands
$j$	Represents number of distinct products available in each ${i}^{th}$ brand
$k$	Represents number of competitive products available corresponding to a particular product i.e. $(n-1)$, when total numbers of products are $n$

### Model development

To explain a modelling framework, all the possible types of customers have been considered that may exist when two products of similar features are available in each product line of two competitive brands in the marketplace.

(Note: For easy representation acronyms have been used i.e. for Maruti Suzuki as M, Hyundai Motors as H. And, Hyundai has its two products under single product line such as H_P1 and H_P2. Similarly, for second competitive brand Maruti has two products M_P1 and M_P2 under one product line.)

The market under consideration can have several products existing simultaneously and hence one can’t be sure of the choice of customers as they can divert from one product to another. Further, all those purchasers who would have adopted Hyundai car (H_P1) but may divert to other products (H_P2, M_P1, M_P2) have been defined. Also, there are chances of deviation of customers from other products (i.e. H_P2, M_P1, M_P2) to H_P1 product. Hence, these customers can be categorized as shown in Table 1:

**Table 1:** Different Customer types

**Customer Type**	**Customer Description**
Type 1	Potential purchasers of H_P1
Type 2	Potential purchasers of H_P2
Type 3	Potential purchasers of M_P1
Type 4	Potential purchasers of M_P2
***Customers who would deviate from one brand (Hyundai) to another brand (Maruti) and/or shift to available but different product designs***	
Type 5	Potential customers of H_P1 who may prefer car of same brand but may prefer different product i.e. H_P1 over H_P2
Type 6	Potential customers of H_P1 who would like to deviate to other brand of similar product i.e. H_P1 over M_P1
Type 7	Potential customers of H_P1 who would prefer other brand along with different product i.e. customers may adopt M_P2 instead of H_P1
Type 8	Potential customers of the H_P2 product, who may prefer other product of same brand and deviate to H_P1
Type 9	Potential customers of the H_P2 product who would prefer different brand and deviate to different product i.e. M_P1 of the other brand
Type 10	Potential customers of H_P2 may prefer another brand of similar product and deviate to M_P2
***Customers who would deviate from one brand (Maruti) to another brand (Hyundai) and/or shift to available but different product designs***	
Type 11	Potential customers of M_P1 prefer same brand but go for another product i.e. H_P1
Type 12	Potential customers of M_P1 who would like to deviate to other brand of the similar product i.e. H_P2
Type 13	Potential customers of product M_P1 who would choose other product of the different brand H i.e. M_P2
Type 14	Potential customers of M_P2 prefer different brand but other product and deviate to H_P1
Type 15	Potential customers of M_P2 may prefer to deviate to other brand of similar product i.e. H_P2
Type 16	Potential customers of M_P2 who may prefer same brand but different product i.e. M_P1

**Fig. 2:**
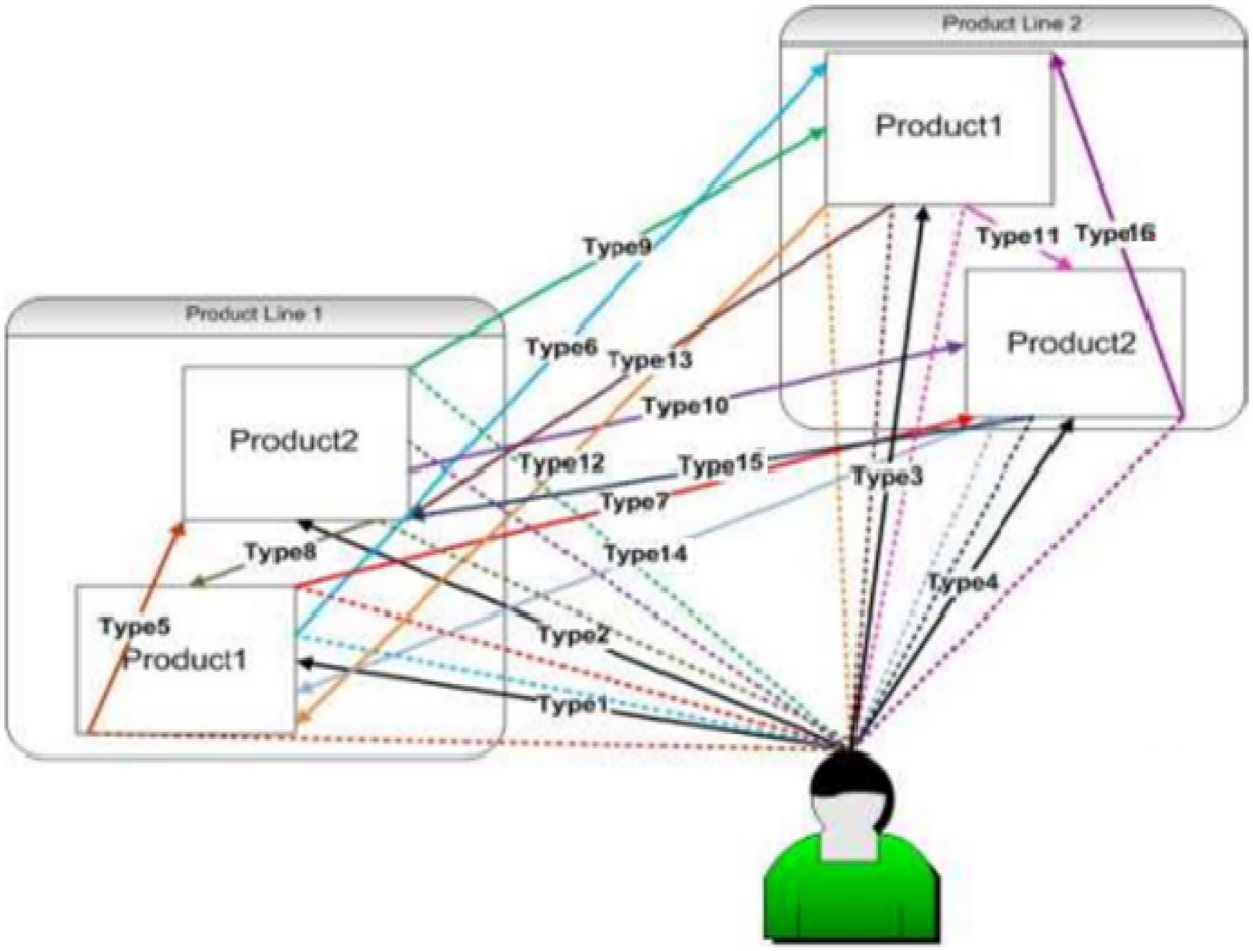
Understanding of Potential Adopters

In order to understand the deviating behavior of the customers we have defined types of adopters from Type 1 to Type 4 are those potential adopters of similar product under single product line but with different brands. Whereas, Type 5 to Type 16 are those expected purchasers who would act as deviators and may deviate to another product with a motive of purchasing best available product in the market. Eventually the role of consumer plays a vital role in deciding which product he/she wishes to choose from. This is very much in line with what researchers like Kapoor and Dwivedi (2020) claimed regarding the sustainable consumption from the consumer’s perspective and with the thought process given by Lowe et al. (2019) wherein Consumers and technology in a changing world have been studied. Categorization of all types of customers can be demonstrated using Fig. 2.

The expression of how aggregated sales of the single Hyundai product line can be segregated into two different but similar products (i.e. H_P1 and H_P2) when available together:

1 $$\begin{aligned}\text{H}\_\text{P1}&=({\lambda }_{1}.{m}_{1}.{F}_{11}(t))-({\alpha }_{1}.{\lambda }_{1}.{m}_{1}.{F}_{11}(t).{F}_{12}(t))-({\alpha }_{2}.{\lambda }_{1}.{m}_{1}.{F}_{11}(t).{F}_{21}(t))\\&-({\alpha }_{3}.{\lambda }_{1}.{m}_{1}.{F}_{11}(t).{F}_{22}(t))+({\delta }_{1}.(1-{\lambda }_{1}).{m}_{1}.{F}_{12}(t).{F}_{11}(t))\\&+({\gamma }_{1}.{\lambda }_{2}.{m}_{2}.{F}_{21}(t).{F}_{11}(t))+({\rho }_{1}.(1-{\lambda }_{2}).{m}_{2}.{F}_{22}(t).{F}_{11}(t))\end{aligned}$$

2 $$\begin{aligned}\text{H}\_\text{P2}&=\left(\left(1-{\lambda }_{1}\right).{m}_{1}.{F}_{12}\left(t\right)\right)-\left({\delta }_{1}.\left(1-{\lambda }_{1}\right).{m}_{1}.{F}_{12}\left(t\right).{F}_{11}\left(t\right)\right)\\&-({\delta }_{2}.\left(1-{\lambda }_{1}\right).{m}_{1}.{F}_{12}(t).{F}_{21}(t))-\left({\delta }_{3}.\left(1-{\lambda }_{1}\right).{m}_{1}.{F}_{12}\left(t\right).{F}_{22}\left(t\right)\right)\\&+({\alpha }_{1}.{\lambda }_{1}.{m}_{1}.{F}_{11}(t).{F}_{12}(t))+({\gamma }_{2}.{\lambda }_{2}.{m}_{2}.{F}_{21}(t).{F}_{12}(t))\\&+({\rho }_{2}.(1-{\lambda }_{2}).{m}_{2}.{F}_{22}(t).{F}_{12}(t))\end{aligned}$$

In Eqs. (1) and (2), where, ${F}_{i,j}(t)=\left[\frac{1-{e}^{-({p}_{ij}+{q}_{ij})t}}{1+\left(\frac{{q}_{ij}}{{p}_{ij}}\right){e}^{-({p}_{ij}+{q}_{ij})t}}\right]$ represent the distribution fraction for the adoption process.*m*_1_are the potential adopters of the aggregated sales of Hyundai cars having single product line consists of two different products. ${\lambda }_{1}$ and $(1-{\lambda }_{1})$ are the rates that classifies the overall potential customers of single product line into respective numbers of distinct products. Hence, $({\lambda }_{1}.{m}_{1}.{F}_{11}(t))$ and $((1-{\lambda }_{1}).{m}_{1}.{F}_{12}(t))$ have become the potential customers of two different products of single product line of brand Hyundai i.e. H_P1 and H_P2.The expected count of customers may get transformed (either increased or decreased) when they get influence from the other products that may satisfied their need better. Here, we have considered two different products which are comparable to each other under two brands. Therefore, potential customers of H_P1 have three different options available to choose from such that either to prefer same brand but consist of different features (H_P2) or to prefer different brand and its products i.e. (M_P1 and M_P2). For this purpose, we have considered ${\alpha }_{1}, {\alpha }_{2}\;and\;{\alpha }_{3}\left(=1-{\alpha }_{1}-{\alpha }_{2}\right)$ competitive factors which can be considered as the rate of diversion from H_P1 to H_P1, M_P1 and M_P2 respectively. Since, these are the deviators as they are deviating from H_P1 to H_P2, M_P1 and M_P2; therefore, we have subtracted them from the total expected sales of H_P1. Similarly, there is likelihood of additions of deviators in some proportions to the overall sales of H_P1 which may come from the other available products such as H_P2, M_P1, and M_P2 with the rate of ${\delta }_{1}, {\gamma }_{1}\;and\;{\rho }_{1}$ as the competitive factors respectively. Therefore, we can say that, potential adopters of H_P1 i.e. ${\lambda }_{1.}{m}_{1.}{F}_{11}(t)$ who would prefer H_P2 with the rate of ${\alpha }_{1.}$ would add up to H_P2 and get subtracted from H_P1 with the rate of ${F}_{12}(t)$. Hence, collectively ${\alpha }_{1.}{\lambda }_{1.}{m}_{1.}{F}_{11}(t{)}_{.}{F}_{12}(t)$ is the factor that acted as the possible count of deviated customers of Type-5. Similarly, we can say for ${\alpha }_{2.}{\lambda }_{1.}{m}_{1.}{F}_{11}(t{)}_{.}{{F}_{2}}_{1}(t)\;{\text{and}}\;{\alpha }_{3.}{\lambda }_{1.}{m}_{1.}{F}_{11}(t{)}_{.}{F}_{22}(t)$ are the deviators who would be considered as Type-6 and Type-7 respectively. Since, these are the terms which are referred to the customers deviating from H_P1 to other products; we have subtracted them from the expression of the overall sales of H_P1. Likewise, $({\delta }_{1.}\left(1-{\lambda }_{1}\right).{m}_{1.}{F}_{12}(t{)}_{.}{F}_{11}(t))$, $({\gamma }_{1.}{\lambda }_{2.}{m}_{2.}{F}_{21}(t{)}_{.}{F}_{11}(t))$ and $({\rho }_{1.}\left(1-{\lambda }_{2}\right).{m}_{2.}{F}_{22}(t{)}_{.}{F}_{11}(t))$ are representing as Type-8, Type-11 and Type-14 of deviators respectively that would be added up in expression of the overall sales of H_P1 and would get subtracted from their respected sales counts such that H_P2, M_P1, and M_ P2. Similarly, we have mentioned similar expression for another product line of Maruti brand follows:

3 $$\begin{aligned}\text{M}\_\text{P1}&=({\lambda }_{2}.{m}_{2}.{F}_{21}(t))-({\gamma }_{1}.{\lambda }_{2}.{m}_{2}.{F}_{21}(t).{F}_{11}(t))-({\gamma }_{2}.{\lambda }_{2}.{m}_{2}.{F}_{21}(t).{F}_{12}(t))\\&-\left({\gamma }_{3}.{\lambda }_{2}.{m}_{2}.{F}_{21}\left(t\right).{F}_{22}\left(t\right)\right)+({\alpha }_{2}.{\lambda }_{1}.{m}_{1}.{F}_{11}(t).{F}_{21}(t))\\&+({\delta }_{2}.(1-{\lambda }_{1}).{m}_{1}.{F}_{12}(t).{F}_{21}(t))+({\rho }_{3}.(1-{\lambda }_{2}).{m}_{2}.{F}_{22}(t).{F}_{21}(t))\end{aligned}$$

4 $$\begin{aligned}\text{M}\_\text{P2}&=((1-{\lambda }_{2}).{m}_{2}.{F}_{22}(t))-({\rho }_{1}.(1-{\lambda }_{2}).{m}_{2}.{F}_{22}(t).{F}_{11}(t))\\&-({\rho }_{2}.(1-{\lambda }_{2}).{m}_{2}.{F}_{22}(t).{F}_{12}(t))-({\rho }_{3}.(1-{\lambda }_{2}).{m}_{2}.{F}_{22}(t).{F}_{21}(t))\\&+({\alpha }_{3}.{\lambda }_{1}.{m}_{1}.{F}_{11}(t).{F}_{22}(t))+({\delta }_{3}.(1-{\lambda }_{1}).{m}_{1}.{F}_{12}(t).{F}_{22}(t))\\&+({\gamma }_{3}.{\lambda }_{2}.{m}_{2}.{F}_{21}(t).{F}_{22}(t))\end{aligned}$$

With the help of the above equations, we can understand the other types of deviators as well. Type-9 are the adopters who may deviate from H_P2 → M_P1 and mathematically represented as ${{\delta }_{2}}_{.}{\left(1-{\lambda }_{1}\right)}_{.}{{m}_{1}}_{.}{F}_{12}(t{)}_{.}{F}_{21}(t)$.Type-10 would fall in the class of ${\delta }_{3.}\left(1-{\lambda }_{1}\right).{m}_{1.}{F}_{12}(t{)}_{.}{F}_{22}(t)$ which would indicate those adopters who have deviated from H_P2 → M_P2. Type-12 are the adopters who are deviated from M_P1 → H_P2 and defined as ${{\gamma }_{2}}_{.}{{\lambda }_{2}}_{.}{{m}_{2}}_{.}{F}_{21}(t{)}_{.}{F}_{12}(t)$. Deviators of Type-13 i.e. ${{\gamma }_{3}}_{.}{{\lambda }_{2}}_{.}{{m}_{2}}_{.}{F}_{21}(t{)}_{.}{F}_{22}(t)$ are those who would deviate from M_P1 → M_P2. Adopters of Type-15 are those who deviated from M_P2 → H_P2 and represented as ${{\rho }_{2}}_{.}{\left(1-{\lambda }_{2}\right)}_{.}{{m}_{2}}_{.}{F}_{22}(t{)}_{.}{F}_{12}(t)$. Adopters of Type-16 are those who deviated from M_P2 → M_P1 and represented as ${{\rho }_{3}}_{.}{\left(1-{\lambda }_{2}\right)}_{.}{{m}_{2}}_{.}{F}_{22}(t{)}_{.}{F}_{21}(t)$.The proposed modelling framework can be generalized up to “x” product consisting of “y” product lines in each brands. In the next section, we have validated these equations and analyzed the deviating behavior customers with respect to the sales of the cars considered.

## Data analysis and numerical illustration

### Numerical illustration

For the validation of our preposition, we have considered aggregated sales of two product lines of different brands (i.e. Hyundai and Maruti) from online sites (Motorbeam 2015; Team-bhp 2021). The products under each product line are slightly different (depending on features, price, etc.) but are comparable to each other given in Table 2 below. This way, the only parameter that differentiates the cars from each other is their brand and any kind of choice made by the customer will imply his/her inclination towards that product because of competitive factor. To validation of our proposed modeling, we have used cars sales data.

**Table 2:** Product Type under Product Line of Hyundai and Maruti

**Attributes**	**Product Type 1**	**Product Type 2**
Car Type	Hatchback	Estate/Hatchback
No. of seats	5-seater	5-seater
Engine Displacement	1 Litre	1.4 Litre
Length	3600 mm	3600–4000 mm

Here, objective is to identify how two product lines with similar attributes and prices are differentiated by the customers’ choice and to predict the sales of all different products that are present in each product line as per customers’ buying behavior in the presence of high competition. Here, first product line includes combined sales of two cars of Hyundai. Similarly, second product line includes combined sales of two products for Maruti. Actual data of these two products lines can be seen in Table 3.

**Table 3:** Sales of Product Lines (P1 and P2)

**Time**	**Product Line Hyundai (in '000)**	**Product Line Maruti (in '000)**
Jan_11	35.407	83.008
Feb_11	65.416	157.81
Mar_11	95.312	239.185
Apr_11	123.928	299.156
May_11	149.493	362.466
Jun_11	174.728	416.888
Jul_11	194.592	464.015
Aug_11	216.106	517.554
Sep_11	246.493	576.6
Oct_11	274.34	612.468
Nov_11	304.202	673.548
Dec_11	329.063	732.794
Jan_12	358.045	810.586
Feb_12	389.617	887.589
Mar_12	423.868	968.328
Apr_12	452.152	1025.12
May_12	478.324	1079.305
Jun_12	502.418	1136.127
Jul_12	524.35	1180.884
Aug_12	546.881	1209.005
Sep_12	572.45	1265.968
Oct_12	602.194	1330.66
Nov_12	632.099	1391.188
Dec_12	655.631	1446.467

Further, for estimating parameters of proposed model, we have used simultaneous non-linear least square methodology and solved it using SAS software package (SAS Institute Inc. 2004). Table 4 is representing the estimated values of diffusion parameters such as external factors (${p}_{ij}$), internal factors (${q}_{ij}$) and competitive factors (${\alpha }_{k},{\delta }_{k}\;{\text{and}}\;{\sigma }_{k}(where\;k=\mathrm{1,2},3)$) of all 4 products i.e. H_P1, H_P2, M_P1 and M_P2 respectively that have been obtained using above proposed Eqs. (1)–(4). And, comparison criteria's of these equations are shown in Table 5.

**Table 4:** Parameter Estimation Results

**Model**	${m}_{\mathrm{1,2}}$ (in ‘000)	${\lambda }_{\mathrm{1,2}}$	${p}_{ij}$	${q}_{ij}$	${\alpha }_{k}$	${\delta }_{k}$	${\gamma }_{k}$	${\sigma }_{k}$
**H_P1 (Eq. (****1****))**	662.402	0.569	0.0211	0.109	-	0.29	0.201	0.202
**H_P2 (Eq. (****2****))**			0.0412	0.101	0.16	-	0.191	0.219
**M_P1 (Eq. (****3****))**	1632.770	0.649	0.0301	0.11	0.45	0.201	-	0.579
**M_P2 (Eq. (****4****))**			0.0221	0.201	0.39	0.509	0.608	-

**Table 5:** Comparison Criteria

**Model**	**SSE**	**MSE**	**Root MSE**	**R-Square**
**PL(H) = H_P1 + H_P2 (Eq. (****1****) + Eq. (****2****))**	6.8259E9	2.9678E8	17227.2	0.9919
**PL(M) = M_P1 + M_P2 (Eq. (****3****) + Eq. (****4****))**	7.171E10	3.1179E9	55837.9	0.9820

**Fig. 3:**
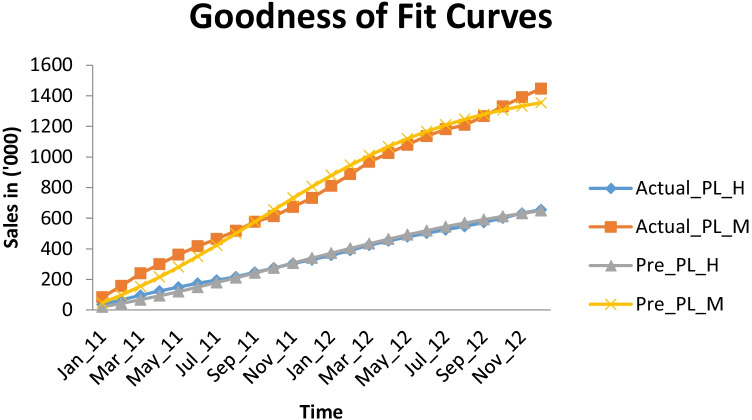
Goodness of Fit Curves

**Fig. 4:**
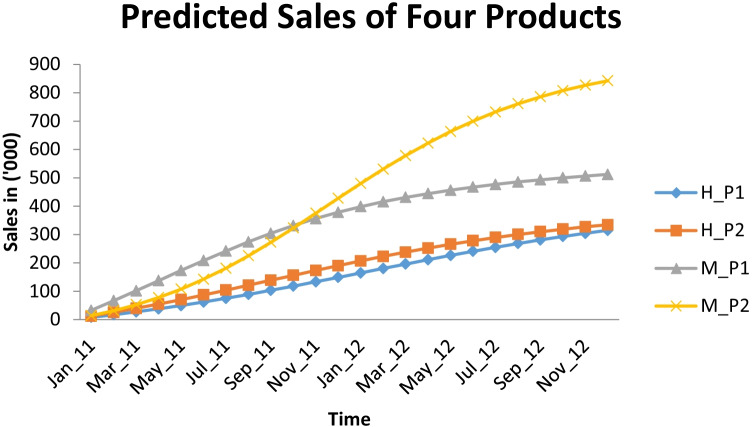
Predicted Sales of Four Products

Using Fig. 3, clearly the graph represents an exceptional fit between actual and predicted values of the two-product line of brands Hyundai and Maruti respectively. And, Fig. 4 represents the predicted sales behavior of four products of different two under two product lines i.e. H_P1, H_P2, M_P1 and M_P2 respectively as per deviating behavior of customers due to competitive factors.

### Results and discussions

Table 4 and Fig. 3 demonstrated that overall potential customers of PL_H and PL_M are observed as ${m}_{1}=662.042\;{\text{and}}\;{ m}_{2}=1632.770$ (in ‘000). This implies that the product mix of cars which hold similar but comparable attribute for Hyundai has total expected number of customers ${m}_{1}=662.042$ and ${m}_{2}=1632.770$ for Maruti. Further, to predict the number of potential customers under each product line that have been classified based on attributes such as car type, number of seat available, type of engine displacement and length, etc. (Table 2). We have made use of parameter ${\lambda }_{j}$ that classifies overall potential customers of one brand into number of distinct product lines available (here we have considered two product lines for each brand i.e. P1 and P2). Using the parameter ${\lambda }_{1}=0.569$ for H_ P1 and $(1-{\lambda }_{1})=0.431$, signifies that more than 50% of the potential customer of Hyundai brand would be H_P1 (i.e. 56.9%) and remaining number of potential customers would belong to H_P2 (43.1%). And similarly, ${\lambda }_{2}=0.649$ and $\left(1-{\lambda }_{2}\right)=0.351$ are representing the expected potential size of Maruti brand for its respective product lines i.e. M_P1 and M_P2. External and internal factors for product H_P2 is highest among all other products which represent that this product is well accepted by the customers.

Also the predicted values of these 4 products demonstrated that sales of product line of brand Maruti are higher than the Hyundai products. Table 5 and Fig. 4, as per the goodness of fit criterion, values of SSE, MSE, Root MSE are low for both product lines (of Hyundai and Maruti) and also the value R-Square corresponding to each of the product line PL_H and PL_M is 0.9919 and .9820 that is both values are approaching to 1 which signifies that the proposed model is well fitted with the actual sales data.

### Theoretical contributions and implications

A new product is a combination of three ingredients i.e. *i) need*- that implies the value or worth for which consumers are ready to pay for; *ii) form-* the actual product which customer consumes; and *iii) technology:* the new or latest techniques that have been used for converting an opportunity into actual form to satisfy customers’ needs. Firms must make a right balance among these tree inputs. A new product may not always be an invention, it generally is embedded with new form in terms of good design, additions of new features, improvisation in existing services or creating qualitative product at lesser cost. Therefore, firms keep updating their products on timely basis and adding into the category of product lines. The foremost objective of the firms is not only to acquire new customers but also to maintain a long-lasting association with their existing customers. The concept of product line thereby comes into picture where existing products and new products can capture new market along with keeping the demands of existing customers satisfied.

This study represents a general scenario where two different brands are offering two similar products with some differentiated features. In general, it is comparatively easy to predict sales when firms have counts of products sold at individual level or at each product wise wherein; they could observe the switching behavior at individual level only. Here, firms have the count of products sold under each product line. And, with the help of the proposed mathematical framework potential customer size for each product is identified. Also, the competitor factors represent what product of which brand is more preferred over other products. When there are only 4 products available in the market, 16 unique and different types of deviating behavior of the customers were observed. Deviating behavior is a common characteristic of the customers as they choose the best among all the possibilities available to them. Therefore, firms must decide which product of which product line has to give more priority over others. Once, deviating nature is clear to the marketer it would be comparatively easy for the firms to define their new strategic goals towards themselves and customers both and can broaden the product line accordingly.

### Managerial implication

The objective of any organization is to design an optimized plan that consists of well-defined strategies along with a perfect mix of products to satisfy customers’ needs. Concept of product line is an abstract plan that provides long tern profits and sustainability to organizations. But on contrary, for every manager it is imperative to analyze and predict diffusion of the related product in conjunction with deviating behavior of all potential customers towards other similar but distinguished products that may belong to same brand or some other brand.

Here, in this study with the help of the automobile example, it is examined that how availability of large numbers of products creates high competition that plays significant role in making best selection of a suitable car. This study is useful for managers in two scenarios such that; it helps managers to identify expected number of customers for respective product lines when overall count of potential customers of a particular brand is available. ${\lambda }_{j}$ is a factor helps to predict how many customers of a brand would be the expected potential customer of its available number of product lines.

Furthermore; it is imperative for managers to examine deviating behavior of customers of each product line of its brand that may deviate to either similar product of the same brand or may deviate to similar product of the other brands. Competitive factors (such as ${\alpha }_{k},{\delta }_{k},{\gamma }_{k}\;{\text{and}}\;{\sigma }_{k}$) for each respective products demonstrate that how much percent of any product can cannibalize the sales of the other products. Hence, this study is serving a proposal to the managers that how one can observe deviating behavior of customers and analyze the sales pattern of products when huge competitions coexist in the marketplace.

### Limitations and future research direction

This study is an attempt to highlight the most important concept i.e. product line that exist in the field of marketing. It developed a mathematical model and discussed a numerical illustration that have been limited to two brands with two products under each brand for the simplicity and providing better understanding of product lines and diffusion modeling. This limitation provides fruitful direction for future research because in real life, automobile industry has various products and competitors that can be study in a generalized modeling framework. Moreover, this mathematical modeling framework can be applied into any sector wherever new opportunities are being offered in terms of new product or new product line or new multi-generational product.

## Conclusion

Every brand and firm is putting extra efforts to serve their customers as best as possible and also overcome the challenges that they face in achieving their set of goals. Being an active participant in the marketplace, a firm has to survive in highly competitive environment. Active participation requires advanced thinking, identifying better opportunities and converting such possibilities into real form by applying latest tools and techniques. For example, now a days; wherein COVID-19 pandemic has impacted each and every business and life of the customers severely; digitization has facilitated a helping hand to the organizations and the customers to keep continue with their normal life as is. In fact, digital technologies and information management have become part of the solution (Dwivedi et al. 2022). Digitization brings a positive change in terms of increasing online shopping so that customers don’t face any challenges, ease in communication and interaction with clients and customers, sharing and storing huge amount of information using cloud functionality and many more. Therefore, firms have to keep updating their current activities / products as per changing market scenarios.

Every new idea takes time to be accepted by customers as it requires to bring customers out from their comfortable mode of using the older technique. A very small fraction of the total set of customers’ always welcome new offering and are ready to try it. Therefore, there is always a risk present for the firms i.e. whether a new product would be liked by the customers or not. Product lines can be a solution for the firms where they can bring additions to their product time to time and can make their customer comfortable with new changes gradually.

This study reflects the competitive impact on the diffusion of any product that has to compete with its own related product of the same brand and also of different brands. Here, aggregated sales of two well-known automobile brands i.e. Hyundai and Maruti Suzuki were considered and calculated their potential adopters, coefficients of innovation and imitation and also the rate of competitive factor of each product that defined the significant deviating behavior of customers with more than 98% of accuracy (${R}^{2}$). Hence, this proposed mathematical modeling is useful to predict sales of product when it is a part of product line and competing with other related brands (products).
